# GINS1 is a prognostic biomarker and correlated with methylation and immune escape in liver hepatocellular carcinoma

**DOI:** 10.3389/fonc.2025.1492599

**Published:** 2025-03-07

**Authors:** Mingchao Liang, Tianqi Lai, Zhen Li, Wei Yu, Mingrong Cao, Nan Yao, Youzhu Hu, Tongzheng Liu, Junjie Liang

**Affiliations:** ^1^ Department of General Surgery, The Affiliated Shunde Hospital, Jinan University, Foshan, China; ^2^ Department of Hepatobiliary Surgery, The First Affiliated Hospital, Jinan University, Guangzhou, China; ^3^ Department of Clinical Medicine, Medical College, Jinan University, Guangzhou, China; ^4^ Department of Pathophysiology, Medical College, Jinan University, Guangzhou, China; ^5^ Guangdong Province Key Laboratory of Pharmacodynamic Constituents of Traditional Chinese Medicine and New Drugs Research, College of Pharmacy, Jinan University, Guangzhou, China

**Keywords:** GINS1, biomarkers, prognosis, methylation, immune escape, liver hepatocellular carcinoma

## Abstract

GINS1 is correlated with a poor prognosis in numerous cancers including liver hepatocellular carcinoma (LIHC). Here, efforts have been made to explore the function and underlying mechanism in LIHC through bioinformatics analysis. The mRNA and protein expression data of GINS1 were downloaded from The Cancer Genome Atlas (TCGA) database, the Clinical Proteomic Tumor Analysis Consortium (CPTAC), the University of Alabama at Birmingham CANcer Data Analysis Portal (UALCAN), and the Human Protein Atlas (HPA) database. Moreover, the protein expression of GINS1 was further substantiated by immunohistochemistry staining from 116 clinical samples. Subsequently, the diagnostic and prognostic role of GINS1 in LIHC patients were determined using receiver operating characteristic (ROC) analysis and the Kaplan-Meier plotter (KM-plotter) database. GeneMANIA and STRING databases were respectively used to construct gene and protein-protein interaction (PPI) networks of GINS1. Enrichment analyses were conducted to investigate the functions of GINS1. To assess the genetic alterations, methylation, and prognostic value, cBioPortal, and MethSurv databases were utilized. Additionally, Tumor Immune Estimation Resource (TIMER), Tumor-Immune System Interaction Database (TISIDB), and Gene Expression Profiling Interactive Analysis (GEPIA) were used to explore the correlation with tumor immune. Differential expression analyses validated the upregulation of GINS1 in LIHC. Furthermore, the prognostic and diagnostic values of GINS1 were substantiated by the ROC curve, Kaplan-Meier plotters, and forest plots. Further enrichment, methylation, and tumor immune microenvironment analyses showed an intimate connection with GINS1. In conclusion, GINS1 which is correlated with methylation and immune escape may predict the prognosis of LIHC.

## 1 Introduction

Liver cancer, the sixth most common cancer, has already become the third leading cause of cancer death worldwide (1). Except for the hepatitis virus, risk factors for liver cancer also include alcohol, smoking, obesity, and diabetes (2, 3). Strikingly, more than 1 million patients will die of liver cancer in 2030 (4), and the metastasis or recurrence rate of liver cancer in 5 years is reported more than 70% (5). In the past decades, comprehensive diagnosis, monitoring, prevention, and treatment techniques have been employed to prevent and suppress this disease efficaciously (6). Notably, molecular targeted therapy has recently been investigated as a breakthrough in liver cancer treatment (7, 8). Therefore, a better comprehension of the molecular mechanisms in liver hepatocellular carcinoma (LIHC) is an essential prerequisite for the development of potential therapeutic strategies.

Interestingly, the tumor microenvironment is a dynamic system and is significantly related to poor prognosis in LIHC patients (9). Besides, tumor progression, invasion, metastasis, and recurrence are profoundly affected by the immune microenvironment of liver cancer (9, 10). Consequently, it is feasible to understand the mechanisms and promote the development of immunotherapy by studying the role of immune-related genes and the immune microenvironment.

GINS1 (PSF1), the constituent of eukaryotic DNA replication machinery, participates in regulating DNA replication (11). Within replisome progression complexes, the GINS tetrameric complex maintains the interaction with the MCM2-7 complex and CDC45 (12, 13). Meanwhile, the GINS tetrameric complex is required for cell growth and chromosomal DNA replication (12, 13). It is widely known that these multiple complexes (CDC45/MCM2-7/GINS) recruit DNA polymerases to regulate the initiation and progression phases of DNA replication (14–16). With high expression in stem cells and progenitor cells related to high proliferation potential, GINS1 promotes tumor growth (17). Reportedly, dysregulation of GINS1 has been demonstrated in association with an ominous prognosis and the progression of cancers (18). Nonetheless, the innate role of GINS1 in LIHC requires further elucidation.

Abnormal DNA methylation in tumors can occur before or after a cell mutation, which regulates gene expression in tumors by recruiting proteins involved in gene suppression or inhibiting the binding of transcription factors to DNA (19, 20). Additionally, it is reported that GINS1 deficiency underlined chronic neutropenia and NK cell deficiency (21). However, the relationship between GINS1, methylation and immune escape is not been fully known yet.

In this study, multiple public databases were employed to validate GINS1 as a novel prognostic biomarker that was correlated with methylation and immune escape in LIHC.

## 2 Materials and methods

### 2.1 TCGA datasets

The TCGA datasets of GINS1 from UCSC Xena (https://tcga.xenahubs.net) were utilized to analyze the expression of GINS1 in 33 types of human cancer. The correlation was evaluated between the expression level of GINS1 and the expression of m6A-related genes (22) in LIHC. In addition, the datasets from TCGA were employed to ascertain the correlation with immune checkpoints in LIHC. The *p*-value<0.05 was considered statistically significant.

### 2.2 Protein expression analysis of GINS1

The protein expression of GINS1 with LIHC datasets was obtained from the CPTAC database (https://proteomics.cancer.gov/data-portal) by using UALCAN (http://ualcan.path.uab.edu). Analysis of protein expression of GINS1 in normal liver tissues and LIHC tissues was conducted with HPA (https://www.proteinatlas.org). Paired samples were collected from 116 patients with pathologically diagnosed LIHC from the First Affiliated Hospital of Jinan University (JNUH) for immunohistochemical staining. The protocol was performed according to the guidelines outlined in the Declaration of Helsinki and approved by the Ethics Committee and Institutional Review Board.

### 2.3 Survival and prognostic analysis

Survival and prognostic analysis was conducted using KM-plotter (http://kmplot.com/analysis). The correlations between GINS1 expression and the overall survival (OS), progression-free survival (PFS), disease specific survival (DSS), and relapse-free survival (RFS) in LIHC were analyzed with associated patient samples separated into two groups by median expression. The hazard ratio (HR) with 95% confidence intervals and log-rank *p*-value were also contained.

### 2.4 Gene-gene interaction, PPI networks, and enrichment analysis

GeneMANIA (http://www.genemania.org) (23) and STRING (https://string-preview.org) were applied to construct gene-gene interaction and PPI networks of GINS1. The GO analysis is a powerful bioinformatics tool to explore on functions of genes in 3 categories, including biological processes (BPs), cellular components (CCs), and molecular functions (MFs). Besides, the top 300 genes most positively and negatively associated with GINS1 from the TCGA database were selected for GO term enrichment and KEGG pathway analyses to investigate the functions of GINS1 in LIHC. GO enrichment and KEGG pathway analyses of co-expression genes were performed by the EnrichGO and EnrichKEGG function in the R package “ClusterProfiler” and visualized by the package “ggplot2”, with the enrichment value set top < 0.05.

### 2.5 Genetic alterations and methylation analysis of GINS1

The cBioPortal (https://www.cbioportal.org) was employed to identify the genetic alterations of GINS1 in 8 datasets, including Peking University, Cancer Cell 2019; INSERM, Cancer Cell 2014; MSK, Clin Cancer Res 2018; INSERM, Nat Genet 2015; MSK, PLOS One 2018; AMC, Hepatology 2014; RIKEN, Nat Genet 2012; TCGA, Firehose Legacy. KM plots for survival outcomes including OS and DFS of GINS1 alterations were contained, and the log-rank test was performed. In addition, MethSurv (https://biit.cs.ut.ee/methsurv), a web portal providing univariable and multivariable survival analysis based on methylation biomarkers using TCGA datasets, was conducted to analyze the methylation sites of GINS1 and evaluated the predictive value of corresponding methylation. The HR with 95% confidence intervals of the overall survival was computed and *p*<0.05 was considered statically significant.

### 2.6 TIMER database analysis

TIMER (http://timer.cistrome.org) database was used for the analysis of the correlation between the expression of GINS1 and immune infiltration. Consequently, the correlation was investigated between the expression of GINS1 and 6 tumor-infiltrating immune cells including B cells, CD8^+^ T cells, CD4^+^ T cells, macrophages, neutrophils, and dendritic cells (DCs). In addition, the next step focused on the correlation with particular immune infiltrating cell subset markers, including markers of CD8^+^ T cells, T cells (general), B cells, monocytes, tumor-associated macrophages (TAMs), M1 macrophages, M2 macrophages, neutrophils, natural killer cells (NKs), dendritic cells, T-helper 1 (Th1) cells, T-helper 2 (Th2) cells, follicular helper T (Tfh) cells, T-helper 17 (Th17) cells, Tregs, and exhausted T cells (24–26). Scatter plots were generated to demonstrate the relationships with the particular gene expression, and Spearman’s correlation and statistical significance were estimated.

### 2.7 TISIDB database analysis

The expression of GINS1 and 28 types of tumor-infiltrating lymphocytes (TILs) across human cancers were identified using the TISIDB (http://cis.hku.hk/TISIDB) database (27). In addition, TISIDB was utilized for analysis of the correlation between the abundance of TILs and the expression of GINS1, and Spearman’s test was conducted.

### 2.8 GEPIA analysis

GEPIA (http://gepia.cancer-pku.cn) is a web portal for cancer and normal gene-expression profiling and interactive analyses based on TCGA and the Genotype-Tissue Expression (GTEx) datasets. It was employed to assess the link with particular markers associated with immune cell infiltration of tumors, including markers of Monocyte, TAMs, M1 macrophages, and M2 macrophages. The Spearman’s test was conducted to determine the correlation coefficient, and statistical significance was estimated.

### 2.9 Statistical analysis

The statistical analysis calculated by the online database in this study was mentioned above. ROC curve was performed to identify the cutoff value of GINS1 using the R software package “pROC”. The heat maps of the correlations between GINS1 and the top 50 positively or negatively associated genes were generated by the R software package “heatmap”. The *p*-value < 0.05 or log-rank *p*-value < 0.05 was considered as statistically significant.

## 3 Results

### 3.1 Differential expression of GINS1 in LIHC

**Figure 1:**
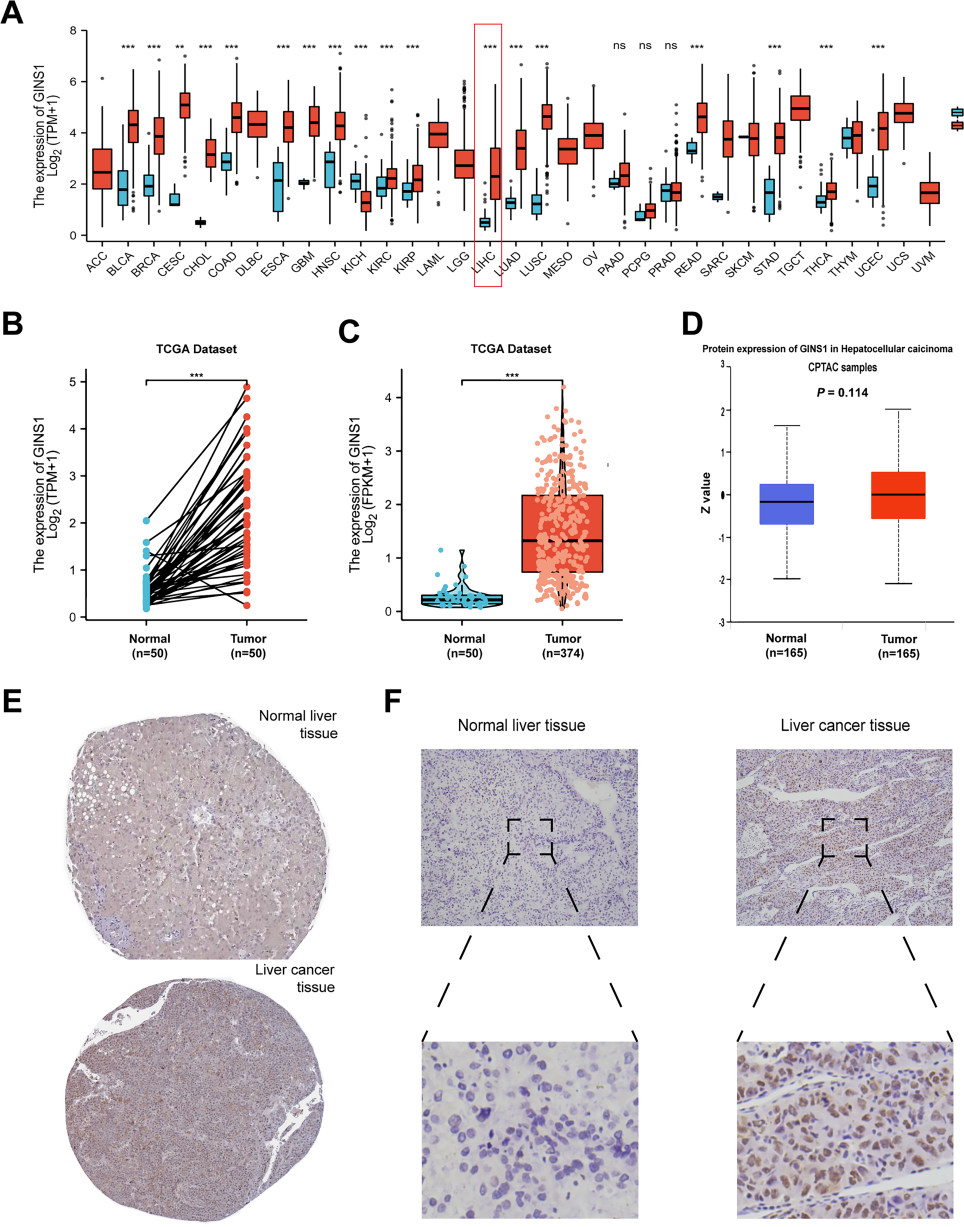
The analysis of GINS1 expression. **(A)** Expression pattern of GINS1 in pan-cancer perspective by TCGA database. **(B)** Expression of GINS1 in 50 LIHC and matched-adjacent normal paired samples. **(C)** Expression of GINS1 in 374 LIHC and 50 normal unpaired samples. **(D)** The protein expression level of GINS1 were analyzed based on CPTAC using UALCAN. **(E)** The protein levels of GINS1 in normal liver tissues and liver cancer tissues (Antibody HPA051185) from the HPA database. **(D)** Immunohistochemical staining of GINS1 in clinical LIHC samples. ** means p<0.01; *** means p<0.001; ns means no significance.

The mRNA expression of GINS1 was investigated across 33 types of different tumors relative to normal tissues with the TCGA database. Compared with normal tissues, the expression of GINS1 was significantly upregulated in tumors including bladder urothelial carcinoma (BLCA), breast invasive carcinoma (BRCA), cervical squamous cell carcinoma (CESC), cholangiocarcinoma (CHOL), colon adenocarcinoma (COAD), esophageal carcinoma (ESCA), glioblastoma (GBM), head and neck squamous cell carcinoma (HNSC), kidney renal clear cell carcinoma (KIRC), kidney renal papillary cell carcinoma(KIRP), liver hepatocellular carcinoma (LIHC), lung adenocarcinoma (LUAD), lung squamous cell carcinoma (LUSC), rectum adenocarcinoma (READ), stomach adenocarcinoma (STAD), thyroid carcinoma (THCA) and uterine corpus endometrial carcinoma (UCEC), but was significantly down-regulated in kidney chromophobe (KICH) (**Figure 1A**). Both paired and unpaired sample analyses revealed that the mRNA expression of GINS1 was significantly higher in LIHC tissues than in the adjacent normal tissues (*p* < 0.001) (**Figures 1B, C**). To explore the protein expression of GINS1, the data in CPTAC using UALCAN were investigated, and no significant difference could be found in normal tissues (*n* = 165, *p* = 0.114) (**Figure 1D**). In addition, immunohistochemical staining in the HPA database and clinical LIHC samples both confirmed that the protein level of GINS1 in LIHC tissues was higher than that in normal tissues (**Figures 1E, F**). These results substantiated upregulated mRNA and protein expression of GINS1 in LIHC tissues.

### 3.2 Correlation between GINS1 expression and clinicopathological parameters of LIHC patients

The Mann-Whitney U-test was performed to determine the correlation between the expression of GINS1 and clinicopathological parameters of LIHC patients. Results revealed that the expression of GINS1 was significantly elevated in patients with higher T classification (*p* = 0.007) and younger age (*p* = 0.020) (**Table 1**). However, other clinicopathological parameters including N and M classification showed no statistically significant association (**Table 1**).

**Table 1:** Clinical characteristics of the patients (TCGA).

Characteristic	Total	Low GINS1	High GINS1	*P*
	N=374	N=187	N=187	
T stage, n (%)				**0.007**
T1	283	107 (28.8%)	76 (20.5%)	
T2	95	41 (11.1%)	54 (14.6%)	
T3	80	30 (8.1%)	50 (13.5%)	
T4	13	6 (1.6%)	7 (1.9%)	
N stage, n (%)				0.624
N0	254	121 (46.9%)	133 (51.6%)	
N1	4	1 (0.4%)	3 (1.2%)	
M stage, n (%)				0.361
M0	268	130 (47.8%)	138 (50.7%)	
M1	4	3 (1.1%)	1 (0.4%)	
Age, median (IQR)	123	64 (54, 70)	59 (51, 67.75)	**0.020**

Statistical significance (*P* < 0.05) is shown in bold.

### 3.3 The diagnostic and prognostic value of GINS1 in LIHC

**Figure 2:**
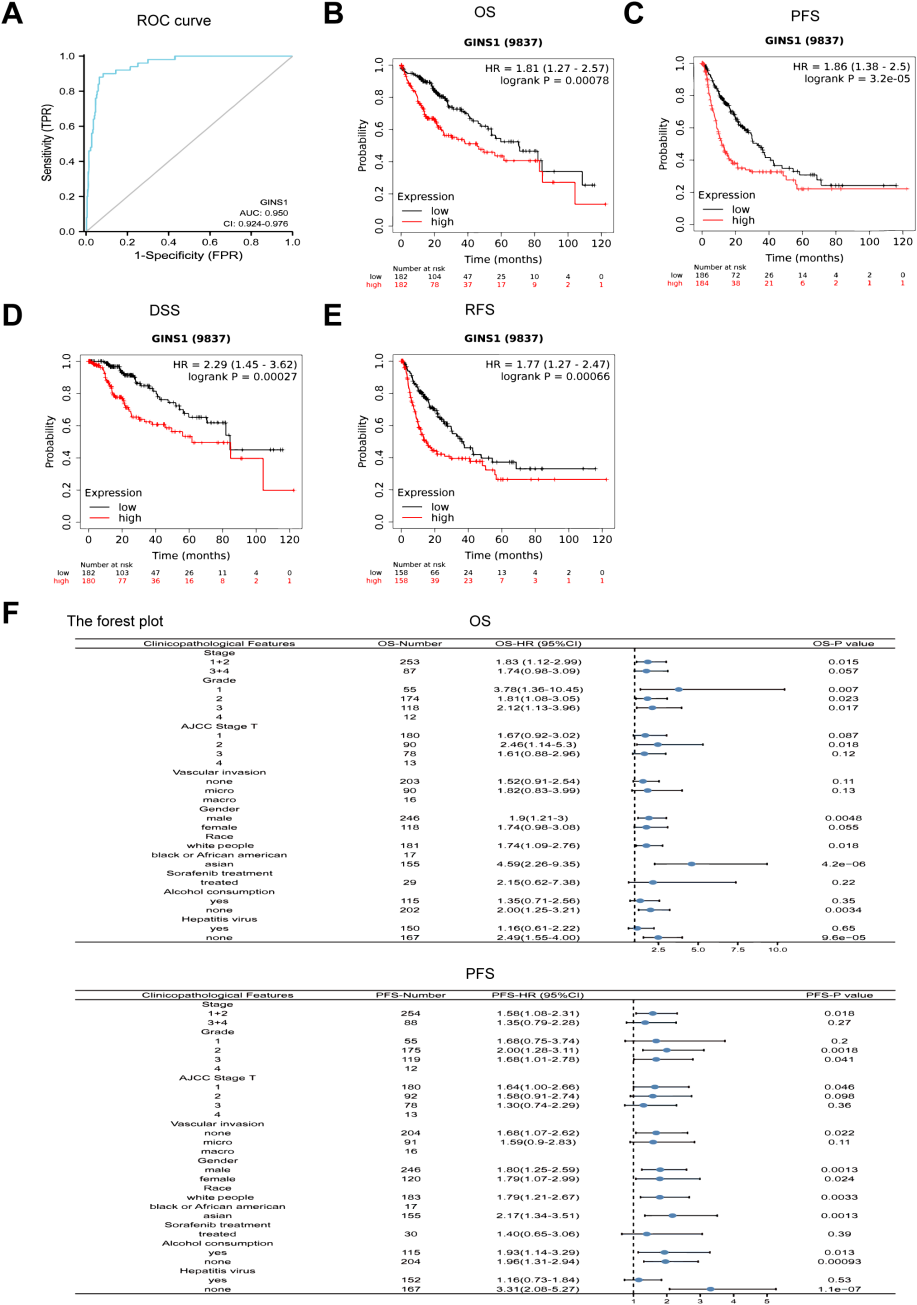
ROC and Kaplan-Meier curves evaluating the prognostic value of GINS1. **(A)** ROC curve of GINS1 in LIHC patients. **(B–E)** Kaplan-Meier analysis of OS, PFS, DSS, and RFS. **(F)** Forest plots of the correlation between GINS1 expression and clinicopathological parameters in LIHC patients. OS, overall survival; PFS, progression-free survival; DSS, disease specific survival; RFS, relapse-free survival.

ROC curve analysis was conducted to ascertain the value of GINS1 in distinguishing LIHC samples from normal samples. Result uncovered that the value of AUC was 0.950 (95% CI: 0.924-0.976) (**Figure 2A**). This result divulged that GINS1 could be considered a potential biomarker for distinguishing LIHC tissues from normal tissues. Consequently, the correlation with prognosis in LIHC patients was assessed by using the KM-plotter. It was divulged that higher expression of GINS1 in LIHC patients was significantly correlated with poorer OS, PFS, DSS, and RFS (all *p* < 0.001) (**Figures 2B-E**). To better understand the prognostic value of GINS1 expression, further effort was made for the correlation with prognosis in LIHC patients based on clinicopathological parameters in the KM-plotter database. In LIHC patients, higher expression of GINS1 was associated with poorer OS in Stage 1 + 2, Grade 1-3, T 1-2, males, white people, Asians, no alcohol consumption, and no hepatitis virus, and poorer PFS in Stage 1 + 2, Grade 2-3, T 1, white people, Asians, no vascular invasion, and no hepatitis virus (**Figure 2F**). Coherently, these results implied that GINS1 could be a promising biomarker to diagnose LIHC and predict the prognosis of LIHC.

### 3.4 Gene-gene interaction, PPI networks, and enrichment analysis of GINS1 in LIHC

**Figure 3:**
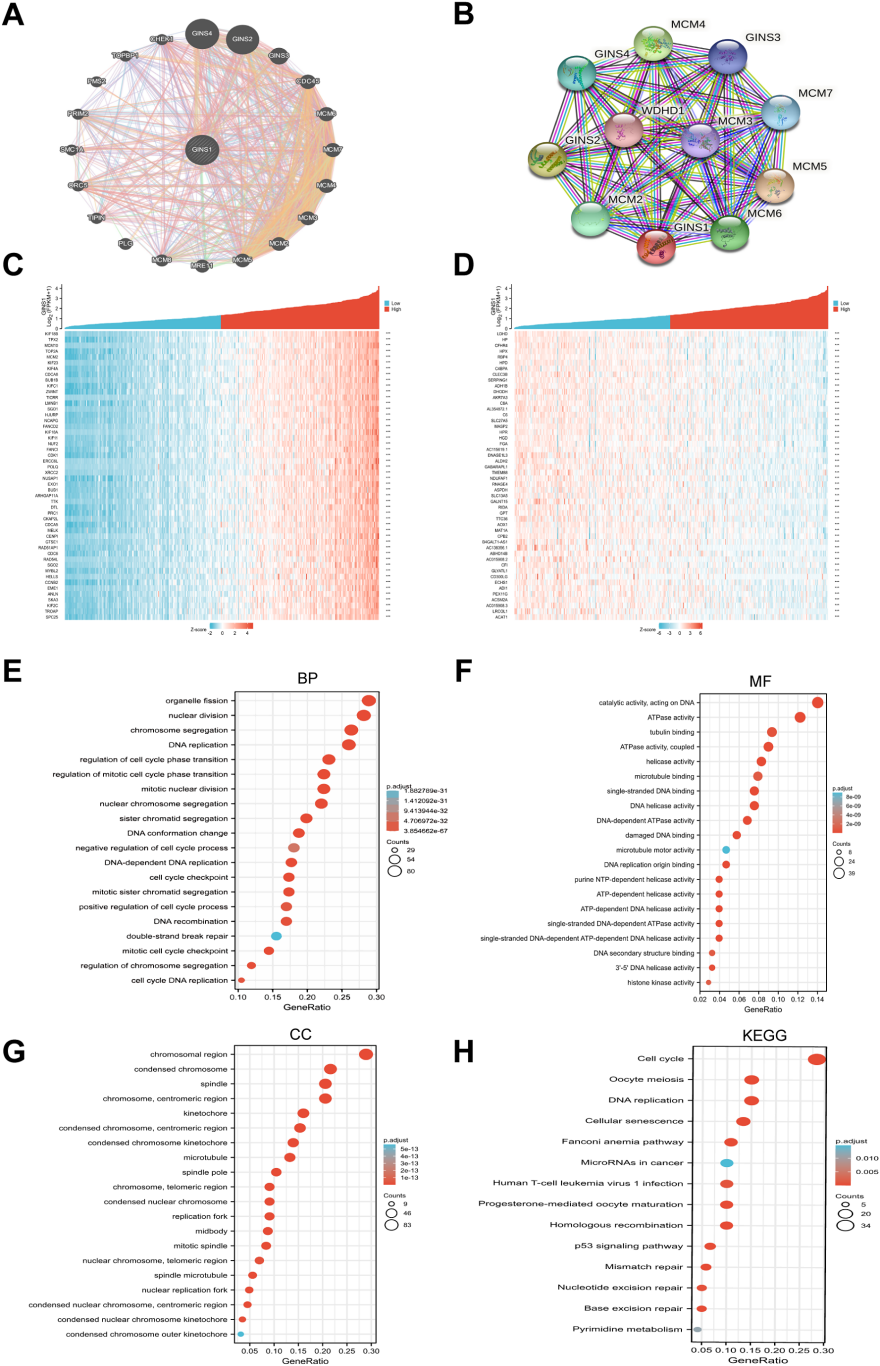
Gene-Gene, PPI network, and functional enrichment analysis of GINS1. **(A)** The gene-gene interaction network of GINS1 by GeneMania. **(B)** The PPI network of GINS1 by STRING. **(C)** Heat maps show the top 50 genes positively correlated with GINS1 in LIHC. **(D)** Heat maps show the top 50 genes negatively correlated with GINS1 in LIHC. **(E–G)** Top 20 enrichment terms in BP, MF, and CC categories in LIHC. **(H)** Top 14 KEGG enrichment pathways in LIHC. BP, biological processe; CC, cellular component; MF, molecular function; KEGG, Kyoto Encyclopedia of Genes and Genomes.

GeneMania and STRING databases were applied to construct gene-gene interaction and PPI networks of GINS1, respectively. As shown in **Figure 3A**, the 20 most frequently altered genes were closely associated with GINS1, including GINS4, GINS2, and GINS3. Functional analysis revealed that these genes were significantly related to DNA replication (**Figure 3A**). Moreover, the PPI networks for GINS1 showed 55 edges and 11 nodes, including MCM5, GINS2, and MCM4 (**Figure 3B**). The co-expression genes in the PPI networks of GINS1 were explored via data mining from the TCGA database. Subsequently, the top 50 genes that were positively and negatively correlated with GINS1 in LIHC were discovered (**Figures 3C, D**). GO enrichment, and KEGG pathway analyses were performed using the top 300 positive related genes. The top 20 significant terms of BPs, MFs, and CCs enrichment analyses were presented (**Figures 3E–G**). In terms of BPs, MFs, and CCs, GINS1 was enriched in the initiation and progression of DNA replication, DNA unwinding and replication, and chromosomal replication processes. Moreover, the top 14 KEGG pathways for GINS1 and related genes were presented (**Figure 3H**). Among these pathways, many DNA replication and cell cycle-related pathways were highly associated with GINS1. Based on the above results, GINS1 was conjectured that it played a certain role in the progression of LIHC and could be a potential therapeutic target of LIHC.

### 3.5 Genetic alteration and methylation analysis of GINS1 in LIHC

**Figure 4:**
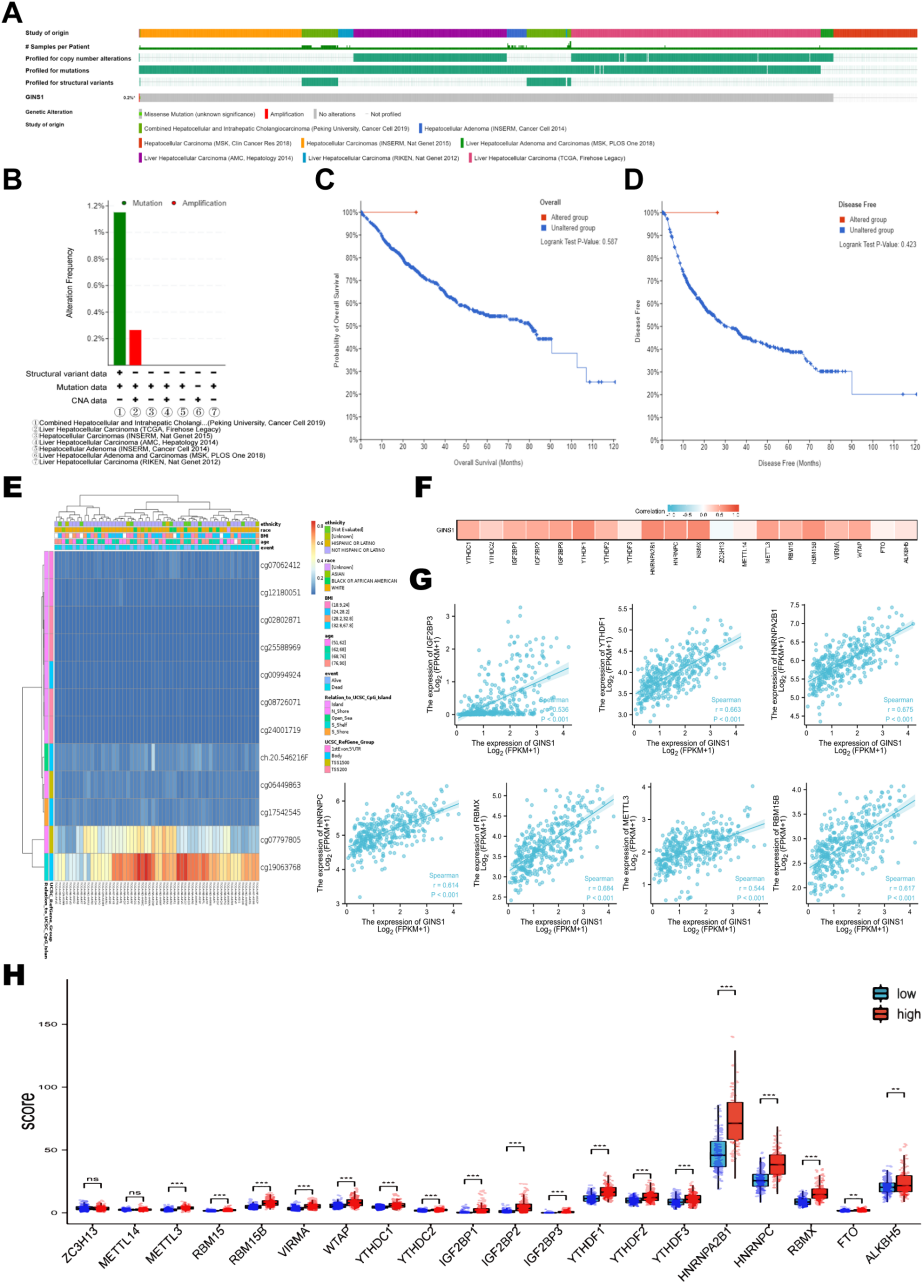
Genetic alteration and methylation analysis of GINS1 in LIHC. **(A)** Genetic alteration of GINS1 in LIHC. **(B)** Summary of GINS1 genetic alteration in LIHC from seven datasets. **(C, D)** Kaplan-Meier plots of OS and DFS in patients with or without genetic alteration of GINS1 in LIHC. **(E)** Visualization of the CpG methylation sites of GINS1 in LIHC. **(F)** The correlation between GINS1 expression and m6A-related genes in LIHC based on TGCA datasets. **(G)** The scatter plots of the correlation between GINS1 and the 7 m6A-related genes with the highest correlation coefficient. **(H)** The differential expression of m6A-related genes in the high and low GINS1 expression groups in LIHC. ** means p<0.01; *** means p<0.001; ns means no significance.

The genetic alteration frequency of GINS1 in LIHC was analyzed using a total of 1245 patients with LIHC from 8 datasets. Firstly, the percentage of GINS1 genetic alteration in LIHC was 0.2% (**Figure 4A**). Besides, the incidence rate of mutation was 1.16% (2/173), while the incidence rate of amplification was 0.27% (1/377) (**Figure 4B**). Meanwhile, the analysis of the KM-plotter found no statistically significant difference between the genetic alteration of GINS1 and OS or PFS, which was probably the result of the insufficient samples (**Figures 4C, D**). In addition, MethSurv was employed to evaluate the DNA methylation sites of GINS1 and the prognostic value of corresponding CpG methylation. 12 methylations of CpG sites were observed, and cg19063768 had the highest DNA methylation (**Figure 4E**). High methylation of cg07062412 was associated with a worse OS in LIHC, while high methylation of cg02802871, cg17542545, cg24001719, ch.20.546216F was associated with a better OS (all *p* < 0.05) (**Table 2**). Next, TCGA datasets were utilized to estimate the correlation with 20 m6A-related genes in LIHC. Results exposed positive correlation of the expression of GINS1 with 17 m6A-related genes in LIHC, including METTL3 (*r* = 0.544), YTHDC1 (*r* = 0.457), YTHDC2 (*r* = 0.271), RBM15 (*r* = 0.431), RBM15B (*r* = 0.617), IGF2BP1 (*r* = 0.442), IGF2BP2 (*r* = 0.489), IGF2BP3 (*r* = 0.536), VIRMA (*r* = 0.395), WTAP (*r* = 0.491), YTHDF1 (*r* = 0.663), YTHDF2 (*r* = 0.408), YTHDF3 (*r* = 0.136), HNRNPA2B1 (*r* = 0.675), HNRNPC (*r* = 0.614), RBMX (*r* = 0.684), and ALKBH5 (*r* = 0.154) (all *p* < 0.05) (**Figure 4F**). The highest correlation coefficient of 7 m6A-related genes was also demonstrated as scatter plots (**Figure 4G**). Notably, 424 LIHC samples were divided into two groups by median expression of GINS1, and higher expression of 18 m6A-related genes was observed in the high GINS1 expression group compared with the low GINS1 expression group (**Figure 4H**). Collectively, these results demonstrated that GINS1 was closely related to genetic alteration and methylation which could cause tumor proliferation and migration in LIHC.

**Table 2:** Effect of hypermethylation level on prognosis in LIHC.

CpG	HR (95%CI)	*P-value*
Body-Island-cg00994924	1.048 (0.739-1.488)	0.79
TSS200- Island- cg02802871	0.607 (0.428-0.861)	**0.0051**
TSS1500- N_Shore-cg06449863	1.493 (0.951-2.344)	0.082
1stExon;5'UTR- Island-cg07062412	1.666 (1.082-2.564)	**0.02**
TSS1500- N_Shore-cg07797805	1.222 (0.86-1.738)	0.26
TSS200- Island-cg08726071	1.204 (0.798-1.815)	0.38
1stExon;5'UTR- Island-cg12180051	1.325 (0.94-1.866)	0.11
Body- S_Shore-cg17542545	0.659 (0.457-0.951)	**0.026**
Body- S_Shelf-cg19063768	0.736 (0.519-1.043)	0.084
TSS200- Island-cg24001719	0.532 (0.371-0.763)	**0.0006**
TSS200- N_Shore-cg25588969	0.867 (0.608-1.237)	0.43
Body- Open_Sea- ch.20.546216F	0.464 (0.305-0.706)	**0.00034**

Statistical significance (*P* < 0.05) is shown in bold.

### 3.6 Correlation between GINS1 expression and immune cell infiltration

**Figure 5:**
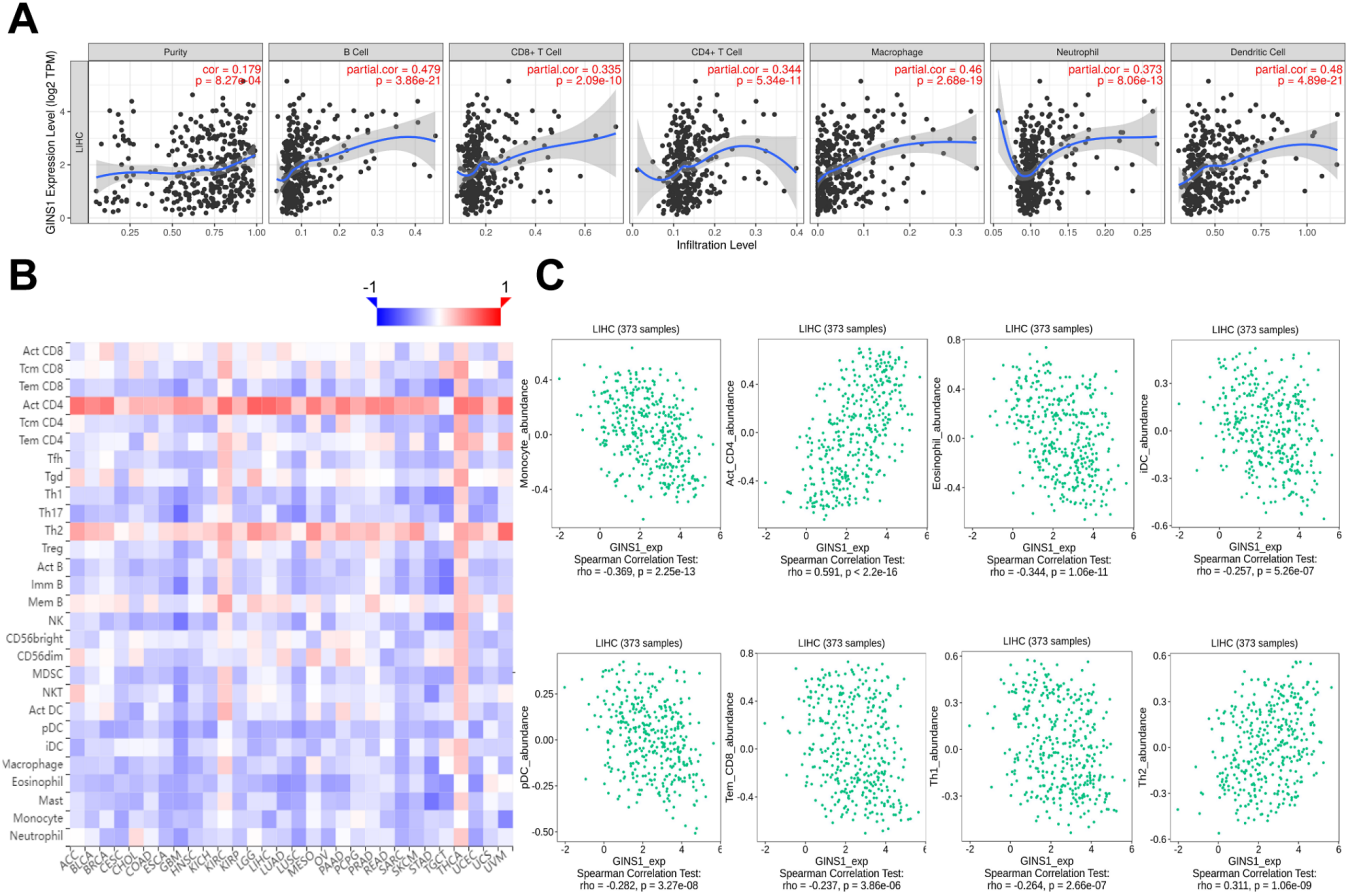
Correlation between GINS1 expression and immune cell infiltration in LIHC. **(A)** The correlation between GINS1 expression and tumor-infiltrating immune cells from TIMER database. **(B)** The correlation between GINS1 expression and 28 types of TILs across human cancers from TISIDB database. **(C)** The correlation between GINS1 expression and the abundance of TILs in LIHC.

Correlation between GINS1 expression and 6 types of tumor-infiltrating immune cells was analyzed using TIMER database. Results showed that the expression of GINS1 was correlated with tumor purity (*r* = 0.179), B cell (*r* = 0.479), CD8^+^ T cell (*r* = 0.335), CD4^+^ T cell (*r* = 0.344), macrophage (*r* = 0.46), neutrophil (*r* = 0.373), dendritic cell (*r* = 0.480) (all *p* < 0.05) (**Figure 5A**). Relations between the expression of GINS1 and 28 types of TILs across human cancers in TISIDB database were shown in **Figure 5B**. Results suggested significant correlation with abundance of monocyte cell (*r* = -0.369), activated CD4 cell (*r* = 0.591), eosinophil cell (*r* = -0.344), immature dendritic cell (*r* = -0.257), plasmacytoid dendritic cell (*r* =-0.282), effector mem-CD8 cell (*r* = -0.237), type 1 T helper cell (*r* = -0.264), type 2 T helper cell (*r* = 0.311) (all *p* < 0.05) (**Figure 5C**). The above results indicated that GINS1 played a specific role in immune infiltration in LIHC.

### 3.7 Correlation between GINS1 expression and immune markers expression

**Figure 6:**
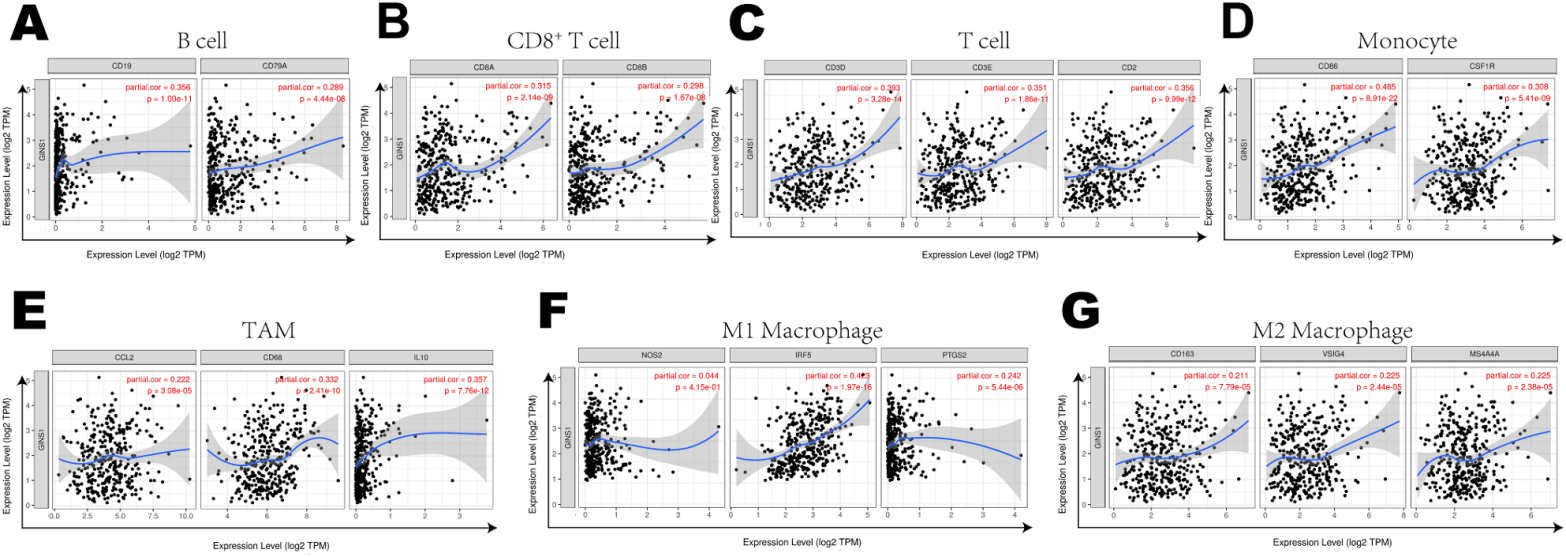
Correlation Between expression of GINS1 and immune markers including **(A–G)** B cell markers (CD19, CD79A), CD8+ T markers (CD8A, CD8B), T cell markers (CD3D, CD3E, CD2), monocyte markers (CD86, CD115), TAM markers (CCL2, CD68, IL10), M1 macrophage markers (IRF5, COX2) and M2 macrophage markers (CD163, VSIG4, MS4A4A) in LIHC.

Further exploration was concentrated on the correlation between the expression of GINS1 and immune marker sets of various immune cells of LIHC in the TIMER and GEPIA databases. Immune marker genes of different immune cells were procured for the correlation analysis with adjustments based on tumor purity (**Table 3**). In particular, the expression of GINS1 was significantly correlated with B cell markers (CD19, CD79A), CD8^+^ T markers (CD8A, CD8B), T cell markers (CD3D, CD3E, CD2), monocyte markers (CD86, CD115), TAM markers (CCL2, CD68, IL10), M1 macrophage markers (IRF5, COX2) and M2 macrophage markers (CD163, VSIG4, MS4A4A) in LIHC (all *p* < 0.0001) (**Figures 6A–G**). In addition, the relationship with these immune markers in LIHC was further investigated using the GEPIA database to confirm the similar association with immune markers of CD8^+^ T cell, T cell, monocytes, B cell, TAM, M1 macrophage, and M2 macrophages (**Table 4**). Surprisingly, the above results substantiated that GINS1 might be capable of regulating the polarization of macrophages in LIHC. Upregulated GINS1 was also associated with increased DC markers, which indicated a closed relationship between GINS1 and tumor DC penetration. Moreover, there was a significant correlation between GINS1 and markers of Treg and exhausted T cells, implying that GINS1 might play an important role in immune escape in LIHC.

**Table 3:** Correlation analysis between GINS1 and related genes and markers of immune cells in TIMER.

Description	Gene markers	LIHC			
		None		Purity	
		Cor	*P-value*	Cor	*P-value*
CD8^+^ T cell	CD8A	0.192	**	0.315	***
	CD8B	0.183	**	0.298	***
T cell (general)	CD3D	0.263	***	0.393	***
	CD3E	0.184	**	0.351	***
	CD2	0.199	**	0.356	***
B cell	CD19	0.273	***	0.356	***
	CD79A	0.163	*	0.289	***
Monocyte	CD86	0.316	***	0.485	***
	CD115 (CSF1R)	0.156	*	0.308	***
TAM	CCL2	0.102	0.050	0.222	***
	CD68	0.229	***	0.332	***
	IL10	0.234	***	0.357	***
M1 Macrophage	INOS (NOS2)	0.037	0.478	0.044	0.415
	IRF5	0.423	***	0.423	***
	COX2 (PTGS2)	0.107	0.040	0.242	***
M2 Macrophage	CD163	0.090	0.082	0.211	***
	VSIG4	0.104	0.045	0.225	***
	MS4A4A	0.087	0.092	0.225	***
Neutrophils	CD66b (CEACAM8)	0.090	0.083	0.123	0.022
	CD11b (ITGAM)	0.331	***	0.436	***
	CCR7	0.095	0.069	0.245	***
Natural killer cell	KIR2DL1	-0.004	0.944	-0.022	0.678
	KIR2DL3	0.178	**	0.226	***
	KIR2DL4	0.199	**	0.235	***
	KIR3DL1	0.009	0.0858	0.027	0.611
	KIR3DL2	0.089	0.088	0.137	0.011
	KIR3DL3	0.037	0.473	0.041	0.447
	KIR2DS4	0.057	0.273	0.049	0.363
Dendritic cell	HLA-DPB1	0.170	*	0.296	***
	HLA-DQB1	0.149	*	0.264	***
	HLA-DRA	0.187	**	0.319	***
	HLA-DPA1	0.160	*	0.297	***
	BCDA-1 (CD1C)	0.134	*	0.235	***
	BDCA-4 (NRP1)	0.257	***	0.288	***
	CD11c (ITGAX)	0.352	***	0.494	***
Th1	T-bet (TBX21)	0.085	0.102	0.201	**
	STAT4	0.271	***	0.346	***
	STAT1	0.397	***	0.457	***
	IFN-γ (IFNG)	0.263	***	0.352	***
	TNF-α (TNF)	0.284	***	0.422	***
Th2	GATA3	0.207	***	0.356	***
	STAT6	0.116	0.025	0.106	0.049
	STAT5A	0.312	***	0.375	***
	IL13	0.129	0.013	0.136	0.011
Tfh	BCL6	0.180	**	0.184	**
	IL21	0.143	*	0.190	**
Th17	STAT3	0.147	*	0.193	**
	IL17A	0.090	0.083	0.107	0.046
Treg	FOXP3	0.219	***	0.306	***
	CCR8	0.401	***	0.509	***
	STAT5B	0.329	***	0.315	***
	TGFβ (TGFB1)	0.268	***	0.371	***
T cell exhaustion	PD-1 (PDCD1)	0.306	***	0.416	***
	CTLA4	0.323	***	0.448	***
	LAG3	0.305	***	0.358	***
	TIM-3 (HAVCR2)	0.315	***	0.487	***
	GZMB	0.073	0.163	0.150	*

LIHC, Liver Hepatocellular Carcinoma; TAM, tumor-correlated macrophage; Tfh, follicular helper T cell; Th, T helper cell; Treg, regulatory T cell; Cor, R value of Spearman’s correlation; None, correlation without adjustment; Purity, correlation adjusted by purity.

*P < 0.01; **P < 0.001; ***P < 0.0001.

**Table 4:** Correlation analysis between GINS1 and related genes and markers of monocyte, TAM and macrophages in GEPIA.

Description	Gene markers	LIHC			
		Tumor		Normal	
		R	*P*	R	*P*
Monocyte	CD86	0.3	***	0.2	0.160
	CD115 (CSF1R)	0.2	***	0.25	0.083
TAM	CCL2	0.069	0.190	0.066	0.650
	CD68	0.22	***	0.19	0.190
	IL10	0.13	0.015	0.25	0.078
M1 Macrophage	INOS (NOS2)	0.00018	1	0.63	***
	IRF5	0.39	***	0.11	0.46
	COX2 (PTGS2)	0.013	0.810	0.025	0.86
M2 Macrophage	CD163	0.15	*	0.081	0.58
	VSIG4	0.19	**	0.089	0.54
	MS4A4A	0.13	*	0.18	0.22

LIHC, Liver Hepatocellular Carcinoma; TAM, tumor-correlated macrophage; Tumor, correlation analysis in tumor tissue of TCGA; Normal, correlation analysis in normal tissue of TCGA.

*P < 0.01; **P < 0.001; ***P < 0.0001.

### 3.8 Correlation between GINS1 expression and immune checkpoints

**Figure 7:**
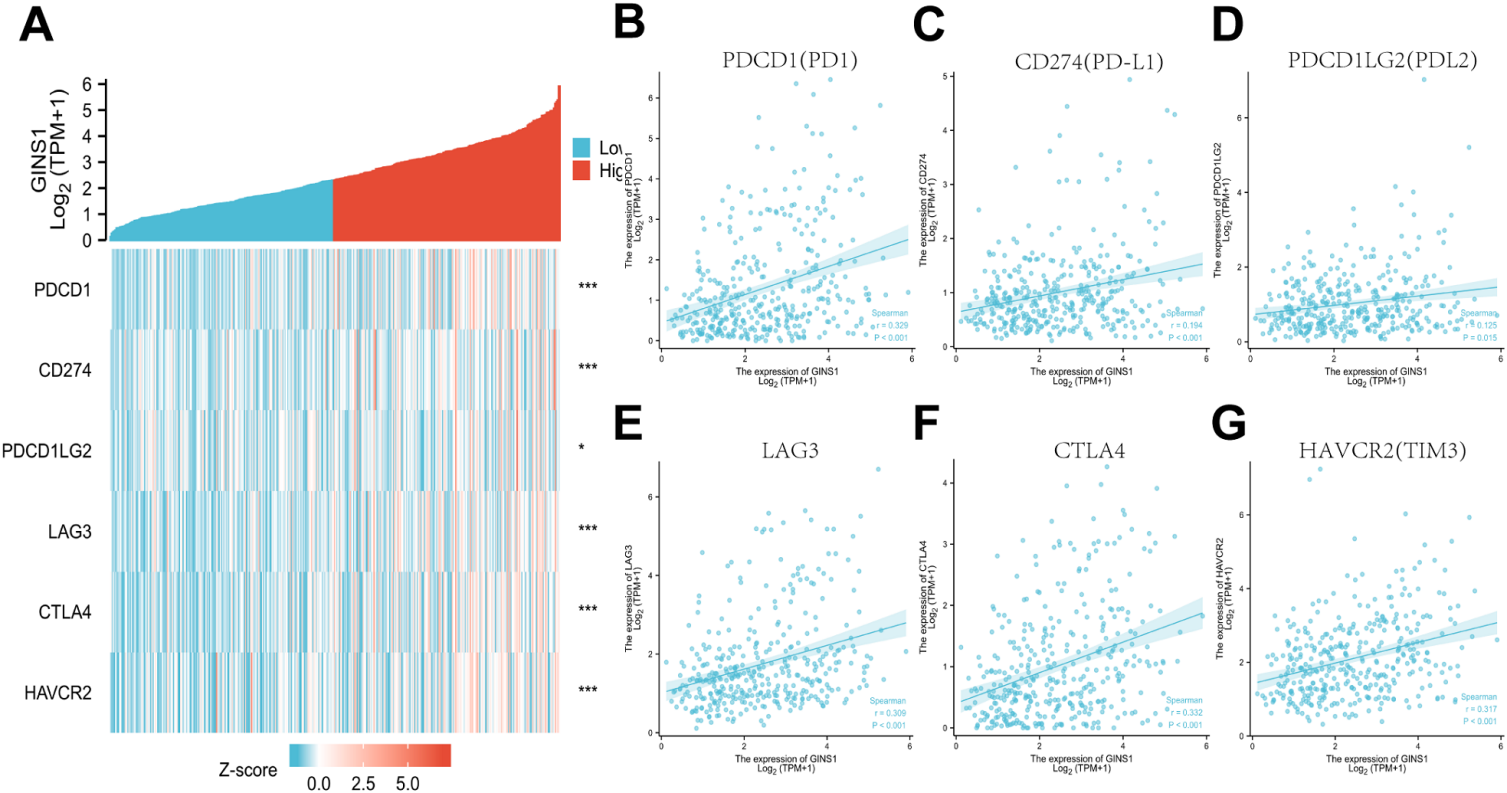
Correlation between GINS1 expression and immune checkpoints in LIHC. **(A)** Heat map of immune checkpoints genes including PDCD1 (PD-1), CD274 (PD-L1), PDCD1LG2 (PD-L2), LAG3, CTLA4, and HAVCR2 (TIM3) based on GINS1 expression. **(B–G)** Scatter plots of the correlation between GINS1 expression and immune checkpoints in LIHC. * means p<0.05; *** means p<0.001.

To further evaluate the association between GINS1 and immune escape, the TCGA datasets were utilized to explore the correlation between GINS1 and immune checkpoint genes including PDCD1 (PD-1), CD274 (PD-L1), PDCD1LG2 (PD-L2), LAG3, CTLA4, and HAVCR2 (TIM3). Heatmap and scatter plots demonstrated significant positive correlations with immune checkpoints above (**Figures 7A–G**), which indicated the unignorable effect of immune escape in GINS1-mediated carcinogenesis of LIHC.

## 4 Discussion

GINS1 is a member of the GINS complex (28) and has been divulged as a component of the eukaryotic DNA replication machinery (11). The oncogenic role of GINS1 in human cancers has been mentioned in many recent studies (28, 29). Moreover, dysregulation of GINS1 has been demonstrated in association with a poor prognosis and the progression of malignant tumors (18, 30). Prior studies discovered that GINS1 could be a target of anlotinib which suppressed the proliferation of synovial sarcoma cells (31). Other trials substantiated that GINS1 might be a target of sorafenib which significantly prolonged the PSF and induced durable responses among patients with progressive, refractory, or symptomatic desmoid tumors (28, 32). However, the role of GINS1 in LIHC has not been fully comprehended. Here, GINS1 was found aberrantly expressed in diverse cancers based on pan-cancer analysis. Our study also confirmed that GINS1 was significantly upregulated in LIHC. High mRNA expression of GINS1 was found positively correlated with a high T stage and younger age. These findings indicated that GINS1 might be a potential biomarker to identify LIHC with poor clinical outcomes. To elucidate the clinical diagnostic value of GINS1 in LIHC, ROC curve analysis was conducted. The results showed that GINS1 had a significantly high AUC value, with 90.0% in sensitivity and 91.7% in specificity. According to the findings above, GINS1 was concluded as a promising diagnostic biomarker to distinguish LIHC from normal liver tissues.

The RAS/RAF/MAPK signaling pathway has been reported to be involved in GINS1-mediated tumor progression (28, 33), and could be regulated by microRNA-340 to suppress the tumorigenic phenotype in melanoma (34). In breast cancer, black rice anthocyanins could suppress metastasis by targeting the RAS/RAF/MAPK pathway (35). In this study, the co-expression analysis showed that the expression of GINS1 was significantly correlated with GINS4, GINS2, GINS3, and MCM2-8. Meanwhile, GO enrichment and KEGG pathway analyses discovered that many pathways related to DNA replication and cell cycle were highly associated with GINS1, including cell cycle, DNA replication, and cellular senescence. The above results substantiated that GINS1 played a certain role in the progression of LIHC and could be a potential therapeutic target of LIHC. However, this needs to be verified by in-depth experiments in the future.

It is widely known that gene mutation is closely related to tumor progression and poor prognosis. In the present study, the percentage of GINS1 genetic alteration in LIHC was only 0.2%, and there was no statistically significant difference between the genetic alteration of GINS1 and OS or PFS. Such results might be the result of insufficient sample size. Aberrant DNA methylation in tumors can occur before or after cellular mutation, and aberrant DNA methylation regulates gene expression in tumors by recruiting proteins involved in gene repression or by inhibiting the binding of transcription factors to DNA (19, 20). Therefore, the relationship was investigated between the DNA methylation levels of GINS1 and the prognosis of LIHC patients. High methylation of cg07062412 was associated with a worse OS in LIHC, however, high methylation of the other 4 CpG was associated with a better OS, which indicated that the DNA methylation level of GINS1 was associated with the prognosis of patients. M6A methylation, which is known as the most important and abundant form of internal modifications in eukaryotic cells, plays a pivotal role in promoting tumor proliferation, migration, and invasion (36). Qi et al. discovered that high expression of m6A-related genes was associated with poor OS of LIHC, except for ZC3H13 (37). Chen et al. found that WTAP was related to m6A modification, contributing to the development of LIHC through the HuR-ETS1-p21/p27 axis (38). The key m6A-related genes METTL3 and METTL14 were reported to be active components of the m6A methyltransferase complex and correlated with tumor proliferation, differentiation, tumorigenesis, invasion, and metastasis (39, 40). In this study, efforts were made to investigate whether GINS1 expression was associated with m6A modification in LIHC. Expression levels of METTL3, RBM15, RBM15B, VIRMA, WTAP, YTHDC1, YTHDC2, IGF2BP1, IGF2BP2, IGF2BP3, YTHDF1, YTHDF2, YTHDF3, HNRNPA2B1, HNRNPC, RBMX, FTO and ALKBH5 increased in the high GINS1 expression group. Thus, we speculated that the GINS1 expression was closely related to m6A modification which promoted the proliferation, migration, and metastasis of LIHC.

The correlation with tumor immune microenvironment in LIHC has not been fully investigated yet. Analysis from the TIMER database unveiled that the expression of GINS1 in LIHC was correlated with several tumor-infiltrating immune cells including B cell, CD8+ T cell, CD4+ T cell, macrophage, neutrophil, and dendritic cell. These results substantiated that GINS1 played a specific role in immune infiltration in LIHC, which could be a potential target for immunotherapy. The next exploration focused on the correlation with immune marker sets of various immune cells of LIHC. Results showed significant correlations with markers of B cell, CD8+ T, T cell, monocyte, TAM, M1 macrophage and M2 macrophage. Moreover, upregulated GINS1 was also associated with increased DC markers. These results revealed that GINS1 might be capable of regulating the polarization of macrophages and tumor DC penetration. There was a significant correlation with markers of Treg and exhausted T cell, and correlation analysis showed significant positive correlations with PDCD1 (PD-1), CD274 (PD-L1), PDCD1LG2 (PD-L2), LAG3, CTLA4, and HAVCR2 (TIM3), indicating the unignorable effect of immune escape in GINS1-mediated carcinogenesis of LIHC.

Admittedly, although the current study includes the verification of immunohistochemical staining experiments, it is only a preliminary exploration in the early stage, and there is a temporary lack of more in-depth experimental verification. However, these research results can preliminarily clarify the feasibility of the research, and are very cost-effective, and provide a theoretical basis for subsequent in-depth research. In the future, we can further verify the relationships between GINS1, methylation and immune microenvironment through relevant experiments, and explore its detailed mechanisms.

## 5 Conclusion

In conclusion, the expression of GINS1 was significantly upregulated in LIHC. The ROC curve, KM-plotter, and forest plot showed the prognostic and diagnostic value of GINS1. Further enrichment, methylation, and tumor immune microenvironment analyses showed an intimate connection with GINS1.

## Data Availability

The raw data supporting the conclusions of this article will be made available by the authors, without undue reservation.
